# Eco-efficiency evaluation of Chinese provincial industrial system: A dynamic hybrid two-stage DEA approach

**DOI:** 10.1371/journal.pone.0272633

**Published:** 2022-08-05

**Authors:** Kai He, Nan Zhu

**Affiliations:** 1 School of Statistics, Southwestern University of Finance and Economics, Chengdu, Sichuan, China; 2 Western Business School, Southwestern University of Finance and Economics, Chengdu, Sichuan, China; South China University of Technology, CHINA

## Abstract

In China, industrial pollution has become an urgent problem for policy makers and enterprise managers. To better support industrial development, we need to determine the effectiveness of policies through efficiency evaluation. China’s provincial industrial system consists of two stages: production and emission reduction. The emission reduction stage is composed of three parallel sub stages: solid waste treatment, waste gas treatment and wastewater treatment. In this process, the treatment capacity of industrial wastewater treatment facilities can be used as carry forward variable, which is not only the desirable output of the previous emission reduction stage, but also the input of the current emission reduction stage. Therefore, this paper proposes a dynamic hybrid two-stage data envelopment analysis (DEA) model for eco-efficiency evaluation of industrial systems, and applies it to a case study of Chinese regional industry. Applying the data collected from 2011 to 2015 to the model, the following conclusions can be drawn: (1) During the whole survey period, the average eco-efficiency was 0.9027. The overall eco-inefficiency of China’s provincial industrial system during the study period is mainly due to low efficiency of solid waste treatment and waste gas treatment. (2) The average eco-efficiency of provincial industrial system increased steadily from 2011 (0.6448) to 2014 (0.6777), but decreased slightly in 2015 (0.5908). (3) The carry forward treatment capacity of industrial wastewater treatment facilities has a remarkable impact on provincial industrial system efficiency scores, especially at the wastewater treatment stage (0.6002 vs 0.3691). (4) Provincial industrial system exists distinct geographical characteristics of low efficiency. This study has important guiding significance for policy makers and enterprise managers who are concerned about industrial pollution control.

## Introduction

Industry is the leading industry of China’s national economy and the inexhaustible driving force for the development of its real economy. Since opening to the outside world, China’s industrial economy has been growing. In the past 20 years, the share of industrial added value in GDP reached 38.2% (data released by China National Bureau of Statistics), and the output of industrial products ranks among the top in the world. However, the growth of China’s industrial economy has paid a huge ecological and environmental cost and consumed a lot of natural resources. This over dependence on resources has brought a series of problems, such as energy depletion, ecological environment deterioration and so on. Statistics show that in 2011, China’s energy consumption increased by nearly 7.3 times from 1977. Total energy consumption increased by nearly 283.241 million tons from 2000 to 2015 (China Energy Statistics Yearbook). The average industrial energy consumption reaches 70% of the total energy consumption [1]. At present, China’s economic transformation and upgrading has become the only way to sustainable development, and the development of industrial economy needs to change from scale accumulation to efficiency and quality improvement. Therefore, it is necessary to re-examine the industrial production system to ensure that the industrial eco-efficiency should be continuously improved while improving the industrial production efficiency.

Extensive industrial economic growth model will bring high resource consumption, heavy environmental burden, and seriously restrict the sustainable development of industrial economy. It has become a consensus that industrial sustainable development can be promoted by improving the eco-efficiency of industrial system [2].

**Fig 1:**
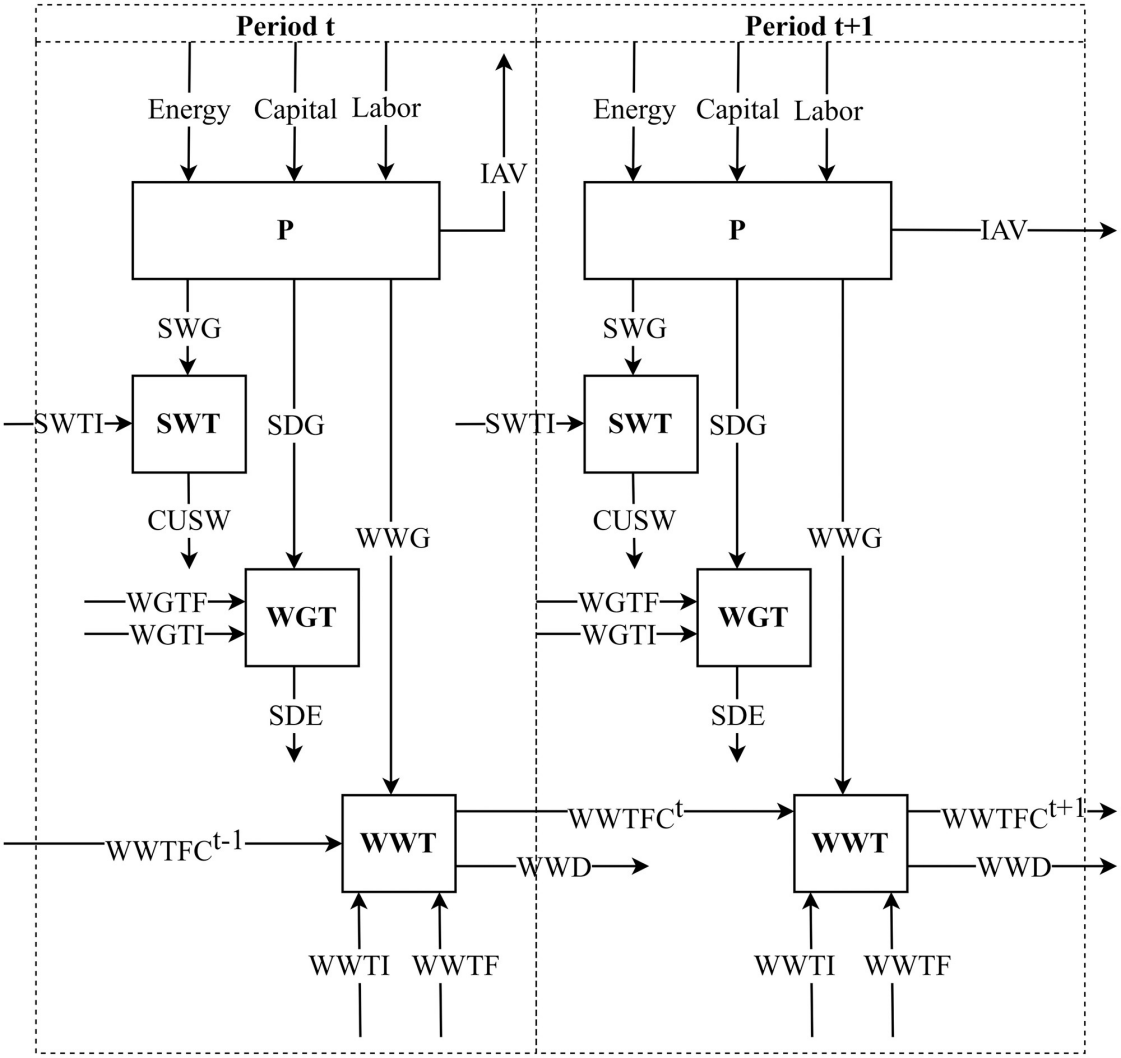
Hybrid two-stage process for provincial industrial systems.

Previous studies mostly regarded the industrial system as a black box, producing industrial pollutant emissions and GDP through input of energy, capital and labor [3]. However, industrial production is often accompanied by the generation of pollutants, some of which will be removed during the treatment process. For example, graphene-based nanomaterial adsorption technology can be used to treat industrial wastewater containing heavy metals, resulting in ultra-low concentration or high quality treated effluent [4]. Specifically, the operational process of China’s industrial sector can be divided into two stages and four linked sub-processes: production (P), wastewater treatment (WWT), waste gas treatment (WGT) and solid waste treatment (SWT). The stage 1 can be called the production stage, and the stage 2 is the emission reduction stage. Stage 2 consists of three parallel industrial pollutant treatment processes, as shown in Fig 1. Therefore, in order to improve the eco-efficiency of the whole system, the efficiency of the four sub-processes must be improved simultaneously. [5] believe that the first stage of industrial production system is usually the production stage, which consumes labor, energy, capital, and other inputs, generates industrial added value (IAV), and emits industrial pollutants. The second stage is the emission reduction stage, through the investment of pollutant treatment funds to reduce the emission of industrial pollutants produced in the production stage. In this paper, industrial pollutants are the output of the first stage and the input of the second stage. The treatment capacity of industrial wastewater treatment facilities in a certain period is the desired output of WWT stage and also the input of WWT stage in the next period. Waste water generation (WWG), solid waste generation (SWG) and sulfur dioxide generation (SDG) are intermediate outputs. Capacity of Industrial wastewater treatment facilities (WWTFC) is a carry forward product. In this article, WWTFC is a desired carry forward variable of the WWT stage of China’s provincial industrial system. Studies have shown that WWTFC changes in a provincial industrial system in a certain period may have a remarkable impact on its wastewater treatment stage efficiency and the eco-efficiency of the industrial system.

[6] found that DEA has been applied extensively in measure industrial performance when reviewing research on industrial system performance. [7] believe that because the two-stage network DEA (NDEA) method can evaluate the production efficiency and pollution treatment efficiency of industrial system at the same time, it has become the main method used to study eco-efficiency. The dynamic network DEA (DNDEA) method can analyze the dynamic effect of carry forward variables on efficiency while opening the black box structure of the system [8]. To better evaluate the eco-efficiency of China’s provincial industrial system, a new dynamic hybrid Two-stage DEA model is established. The method think over the dynamic effects of four stages and a carry forward variable (WWTFC) simultaneously from a multi-stage angle.

The main innovations of this paper are summarized as follows. Firstly, this study considers the dynamic impact of carry forward variable WWTFC on the operation efficiency of provincial industrial system. Secondly, a new slacks-based measure (SBM) dynamic hybrid two-stage model is established to calculate the eco-efficiency, stage efficiency, period efficiency and period stage efficiency, and determine the root cause of the low efficiency of provincial industrial system. Thirdly, the provincial industrial system is divided into Eastern, Central, Western and Northeast regions, which provides useful information for the study of the geographical differences in the efficiency of China’s provincial industrial system.

The remaining chapters of this paper are organized as follows: review the relevant research on industrial system efficiency is carried out in Literature Review. In Methodology, a new dynamic hybrid two-stage SBM model is proposed. The Results applies the proposed method to evaluate the efficiency of China’s provincial industrial system. Relevant discussions are carried out in Discussion. Come to the conclusion in Conclusion.

## Literature review

DEA is a common method to measure eco-efficiency [9]. [10] first proposed using DEA to measure eco-efficiency. Later, more and more researchers use DEA model to study eco-efficiency from different perspectives, such as [11–14] and so on. In the production framework, early researchers used the traditional DEA model to analyze eco-efficiency. The input is energy consumption and the desired output is GDP, but ignored the undesired output [15]. Most of the early studies used the basic DEA model, which did not consider the internal structure when measuring production efficiency, energy was the input and industrial added value was the desired output. This method cannot effectively identify the causes of inefficiency, often resulting in overestimation of efficiency. As [16] believes, NDEA model can solve the problem of efficiency distinction between production stage and pollutant treatment stage, which can not be solved by traditional DEA model [17]. Based on this, Later, more and more studies began to use NDEA model to evaluate eco-efficiency [18].

[19] proposed a NSBM method to evaluate the environmental efficiency of China’s industrial system. The two-stage model is a case of NDEA model. Considering the particularity of different stages, NDEA model can only provide the eco-efficiency of the whole system, but cannot effectively distinguish the efficiency of different stages. However, two-stage NDEA model can simultaneously evaluate the production efficiency and pollution control efficiency in the industrial system, and also investigate the eco-efficiency of the system [20]. Using a two-stage super-NDEA approach, [21] studied the overall efficiency and eco-efficiency of each sub-stage of Chinese industrial system from 2004 to 2015. [22] studied the efficiency of provincial industrial sectors in China using a two-stage NDEA approach that considers shared inputs and recovery of resources from undesired outputs.

However, these studies did not take into account the dynamics of the multistage scenario. By using the two-stage DNDEA model, system efficiency and stage efficiency can be obtained, and the change trend of efficiency with period can be analyzed. [23] proposed the Malmquist-based energy saving and emission reduction performance indicator, which is used to evaluate the performance changes of energy use and pollutant emissions over time in more than 200 Chinese cities. [24] applies DNSBM method to analyze environmental efficiency and force of Chinese electric power system and its provincial administrative divisions. These studies considered the dynamic effect of efficiency, but did not consider its dynamic effect on efficiency in the presence of carry forward variables. [25] used the two-stage DDEA method to study the provincial industrial system of China, considering dynamic effects of the two-stage process and the carry forward CIT. However, in the treatment stage, he did not consider that different industrial pollutants may have different treatment processes, so separate studies are needed to more accurately measure the efficiency of the industrial system. [20] divides the industrial production system into energy consumption stage, wastewater treatment stage and waste gas treatment stage, and uses NSBM to conduct an study on Chinese industrial efficiency during 2011–2015. Although [20]’s study considered the wastewater treatment stage and the waste gas treatment stage separately, it did not consider the dynamic effect. A similar study was done by [26].

The dynamic factors of carry forward WWTFC and the differences of different pollutant treatment processes were not considered in all the above studies. Therefore, the existing measurement methods of industrial system eco-efficiency may not be suitable for dynamic evaluation of Chinese provincial industrial system efficiency with two-stage multi-sub process. This is because intermediates and carry forward variables are ignored. An industrial system is evaluated as inefficient, but it is impossible to know which internal stage caused this inefficiency or whether it was caused by carry forward products. Therefore, better methods are needed to evaluates the eco-efficiency of dynamic two-stage multi-sub stages in Chinese provincial industrial systems.

## Methodology

**Fig 2:**
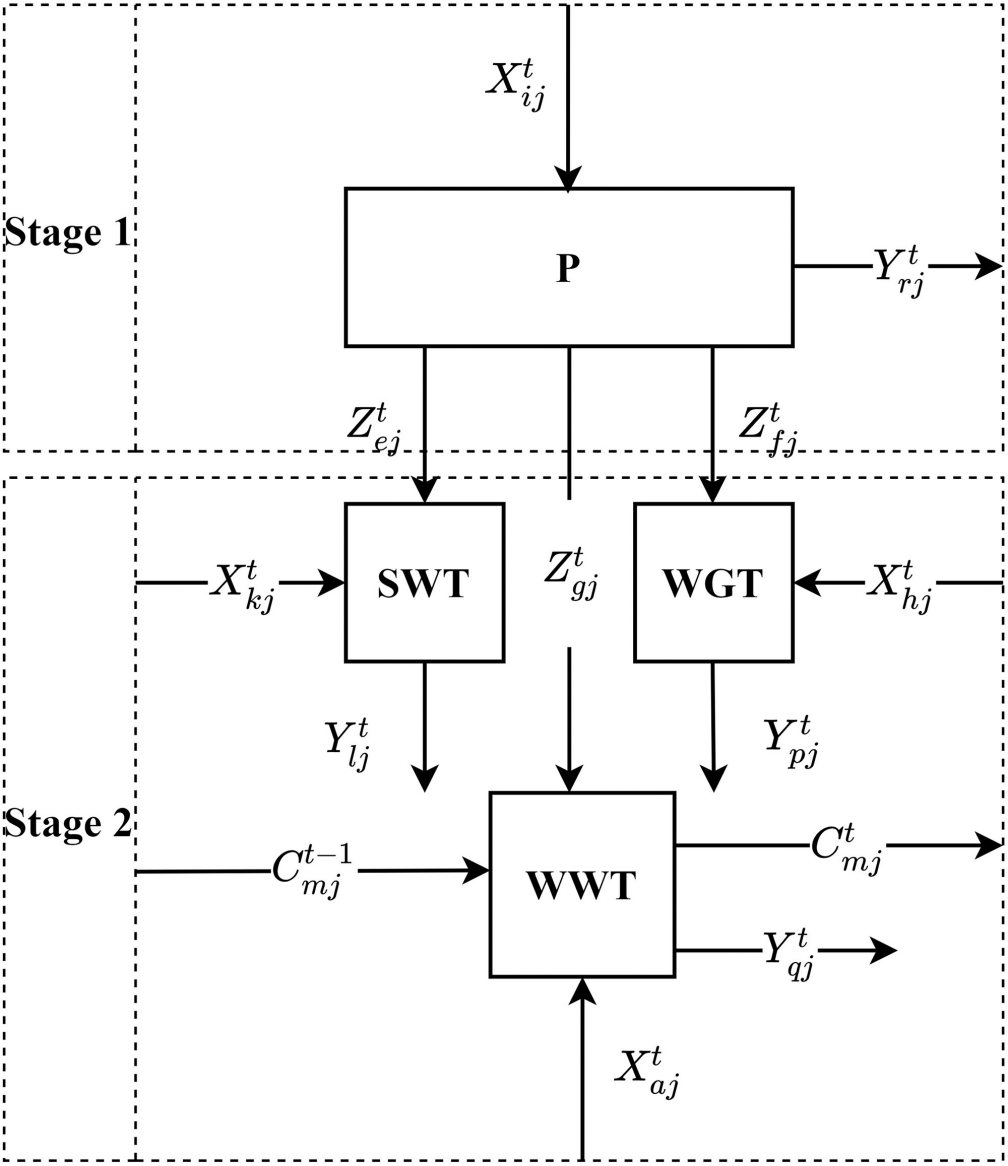
Structure of Chinese industrial system in a specific period t.

The operation process of a provincial industrial system (i.e. DMU) in a certain time t is a two-stage network system. The stage 1 is P stage, and the stage 2 is composed of three parallel sub-stages, namely WWT stage, SWT stage and WGT stage (Fig 1). This creates a hybrid two-stage structure. To facilitate modeling, the structure of Chinese provincial industrial system can be described in a more common form, see Fig 2.

In Fig 2, a *DMU*_*j*_(*j* = 1, 2, …, *n*) to be evaluated during a specific period *t*(*t* = 1, 2, …, *T*), $X_{ij}^{t}\left(i=1,2,\ldots ,I\right)$ are used to produce the desired output $Y_{rj}^{t}\left(r=1,2,\ldots ,R\right)$, and the intermediate products $Z_{ej}^{t}\left(e=1,2,\ldots ,E\right)$, $Z_{fj}^{t}\left(f=1,2,\ldots ,F\right)$ and $Z_{gj}^{t}\left(g=1,2,\ldots ,G\right)$ at the P stage. The P stage produces the intermediate products $Z_{ej}^{t}$, $Z_{fj}^{t}$ and $Z_{gj}^{t}$ for the SWT stage, WGT stage and WWT stage to use, respectively. $X_{kj}^{t}\left(k=1,2,\ldots ,K\right)$ and $Z_{ej}^{t}$ are used to produce the desirable output $Y_{lj}^{t}\left(l=1,2,\ldots ,L\right)$ at the SWT stage. In the WGT stage, the input $X_{hj}^{t}\left(h=1,2,\ldots ,H\right)$ and the intermediate products $Z_{fj}^{t}$ are used to produce undesirable output $Y_{pj}^{t}\left(p=1,2,\ldots ,P\right)$. While input $X_{aj}^{t}\left(a=1,2,\ldots ,A\right)$, intermediate product $Z_{gj}^{t}$ and the carry forward products $C_{mj}^{t-1}\left(m=1,2,\ldots ,M\right)$ of the previous period are used to produce the undesirable output $Y_{qj}^{t}\left(q=1,2,\ldots ,Q\right)$ and the carry forward products $C_{mj}^{t}\left(m=1,2,\ldots ,M\right)$ at the WWT stage.

### Production possibility set

To facilitate modeling, we first make the following assumptions: (1) In real production activities, desired output and undesired output are always produced simultaneously. Therefore, desirable output has strong disposable, while undesirable intermediate outputs are weakly disposable [27, 28]. (2) The carry forward product *C* is fixed at the observation level and is not affected by the technology of the current period t. See [8] for a similar assumptions. Follow [29]’s approach, the production possibility set $PPS^{t}=\{X_{i}^{t},Y_{r}^{t},Z_{k}^{t},X_{k}^{t},Y_{l}^{t},Z_{f}^{t},X_{h}^{t},Y_{p}^{t},Z_{g}^{t},C_{m}^{t-1},X_{a}^{t},C_{m}^{t},Y_{q}^{t}\}\left(t=1,2,\ldots ,T\right)$ established as follows

$$\begin{array}{c} P\;\text{stage}\{\begin{array}{c} \sum_{j=1}^{n}\lambda_{j}^{t}X_{ij}^{t}\leq X_{i}^{t},\forall i,t,\\ \sum_{j=1}^{n}\lambda_{j}^{t}Y_{rj}^{t}\geq Y_{r}^{t},\forall r,t,\\ \sum_{j=1}^{n}\lambda_{j}^{t}Z_{ej}^{t}=Z_{e}^{t},\forall e,t,\\ \begin{array}{c} \sum_{j=1}^{n}\lambda_{j}^{t}Z_{fj}^{t}=Z_{f}^{t},\forall f,t,\\ \sum_{j=1}^{n}\lambda_{j}^{t}Z_{gj}^{t}=Z_{g}^{t},\forall g,t,\\ \lambda_{j}^{t}\geq 0,\forall j,t,\\ \sum_{j=1}^{n}\lambda_{j}^{t}=1,\forall t; \end{array} \end{array} \end{array}$$ (1)

$$\begin{array}{c} \text{SWT}\;\text{stage}\{\begin{array}{c} \sum_{j=1}^{n}\gamma_{j}^{t}Z_{ej}^{t}=Z_{e}^{t},\forall e,t,\\ \sum_{j=1}^{n}\gamma_{j}^{t}X_{kj}^{t}\leq X_{k}^{t},\forall k,t,\\ \sum_{j=1}^{n}\gamma_{j}^{t}Y_{lj}^{t}\geq Y_{l}^{t},\forall l,t,\\ \begin{array}{c} \gamma_{j}^{t}\geq 0,\forall j,t,\\ \sum_{j=1}^{n}\gamma_{j}^{t}=1,\forall t; \end{array} \end{array} \end{array}$$ (2)

$$\begin{array}{c} \text{WGT}\;\text{stage}\{\begin{array}{c} \sum_{j=1}^{n}\eta_{j}^{t}Z_{fj}^{t}=Z_{f}^{t},\forall f,t,\\ \sum_{j=1}^{n}\eta_{j}^{t}X_{hj}^{t}\leq X_{h}^{t},\forall h,t,\\ \sum_{j=1}^{n}\eta_{j}^{t}Y_{pj}^{t}\leq Y_{p}^{t},\forall p,t,\\ \begin{array}{c} \eta_{j}^{t}\geq 0,\forall j,t,\\ \sum_{j=1}^{n}\eta_{j}^{t}=1,\forall t; \end{array} \end{array} \end{array}$$ (3)

$$\begin{array}{c} \text{WWT}\;\text{stage}\{\begin{array}{c} \sum_{j=1}^{n}\mu_{j}^{t}Z_{gj}^{t}=Z_{g}^{t},\forall g,t,\\ \sum_{j=1}^{n}\mu_{j}^{t}C_{mj}^{t-1}=C_{m}^{t-1},\forall m,t,\\ \sum_{j=1}^{n}\mu_{j}^{t}X_{aj}^{t}\leq X_{a}^{t},\forall a,t,\\ \begin{array}{c} \sum_{j=1}^{n}\mu_{j}^{t}C_{mj}^{t}\geq C_{m}^{t},\forall m,t,\\ \sum_{j=1}^{n}\mu_{j}^{t}Y_{qj}^{t}\leq Y_{q}^{t},\forall q,t\\ \mu_{j}^{t}\geq 0,\forall j,t,\\ \sum_{j=1}^{n}\mu_{j}^{t}=1,\forall t. \end{array} \end{array} \end{array}$$ (4)

For the *PPS*^*t*^, $\lambda_{j}^{t},\gamma_{j}^{t},\eta_{j}^{t}$ and $\mu_{j}^{t}\left(t=1,2,\ldots ,T\right)$ represent the intensity vectors of the corresponding stages during period t, respectively. In production economics, due to the influence of fixed inputs, returns to scale usually increase in the early stage of production, when the number of variable inputs is relatively small. As the number of variable inputs increases, the return to scale gradually decreases, becomes constant, and finally decreases [30]. Based on this, variable return to scale (VRS) model is selected in this paper, which can be more consistent with the actual production situation. If $\sum_{j=1}^{n}\lambda_{j}^{t}=1,\sum_{j=1}^{n}\gamma_{j}^{t}=1,\sum_{j=1}^{n}\eta_{j}^{t}=1$ and $\sum_{j=1}^{n}\mu_{j}^{t}=1$ are removed from Eqs (1), (2), (3) and (4), *PPS*^*t*^ will be constant return to scale (CRS).

Since stage 1 and stage 2 are usually continuous in production activities, the linking relationship between stages can be described as [25]

$$\begin{array}{c} \sum_{j=1}^{n}\lambda_{j}^{t}Z_{ej}^{t}=\sum_{j=1}^{n}\gamma_{j}^{t}Z_{ej}^{t},\forall e,t, \end{array}$$ (5)

$$\begin{array}{c} \sum_{j=1}^{n}\lambda_{j}^{t}Z_{fj}^{t}=\sum_{j=1}^{n}\eta_{j}^{t}Z_{fj}^{t},\forall f,t, \end{array}$$ (6)

$$\begin{array}{c} \sum_{j=1}^{n}\lambda_{j}^{t}Z_{gj}^{t}=\sum_{j=1}^{n}\mu_{j}^{t}Z_{gj}^{t}\forall g,t. \end{array}$$ (7)

This paper links different periods by carry forward products, and the formula can be described as follows

$$\begin{array}{c} \sum_{j=1}^{n}\mu_{j}^{t}C_{mj}^{t-1}=\sum_{j=1}^{n}\mu_{j}^{t-1}C_{mj}^{t-1},\forall m,t. \end{array}$$ (8)

### Proposed model

The non-radial measure SBM can identify the causes of inefficiencies in various industrial systems by effectively dealing with input excess and output shortfall [31]. Therefore, this section constructs a dynamic hybrid two-stage model based on SBM to evaluate the eco-efficiency of Chinese provincial industrial system. According to the production possibility set constructed in the previous section, the following models can be established to solve the eco-efficiency, stage efficiency, period efficiency and period stage efficiency of provincial industrial system, respectively.

$$\begin{array}{c} \theta_{o}=\text{min}\frac{\sum_{t=1}^{T}\alpha^{t}[\begin{array}{c} \beta_{1}(1-\frac{1}{I}\sum_{i=1}^{I}\frac{s_{i}^{t-}}{X_{io}^{t}})+\beta_{2}(1-\frac{1}{K}\sum_{k=1}^{K}\frac{s_{k}^{t-}}{X_{ko}^{t}})\\ +\beta_{3}(1-\frac{1}{H+P}(\sum_{h=1}^{H}\frac{s_{h}^{t-}}{X_{ho}^{t}}+\sum_{p=1}^{P}\frac{s_{p}^{t-}}{Y_{po}^{t}}))\\ +\beta_{4}(1-\frac{1}{A+Q}(\sum_{a=1}^{A}\frac{s_{a}^{t-}}{X_{ao}^{t}}+\sum_{q=1}^{Q}\frac{s_{q}^{t-}}{Y_{qo}^{t}})) \end{array}]}{\sum_{t=1}^{T}\alpha^{t}[\beta_{1}(1+\frac{1}{R}\sum_{r=1}^{R}\frac{s_{r}^{t+}}{Y_{ro}^{t}})+\beta_{2}(1+\frac{1}{L}\sum_{l=1}^{L}\frac{s_{l}^{t+}}{Y_{lo}^{t}})+\beta_{4}(1+\frac{1}{M}\sum_{m=1}^{M}\frac{s_{m}^{t+}}{C_{mo}^{t}})]} \end{array}$$ (9)

$$\begin{array}{c} P\;\text{stage}\{\begin{array}{c} \sum_{j=1}^{n}\lambda_{j}^{t}X_{ij}^{t}=X_{io}^{t}-s_{i}^{t-},\forall i,t,\\ \sum_{j=1}^{n}\lambda_{j}^{t}Y_{rj}^{t}=Y_{ro}^{t}+s_{r}^{t+},\forall r,t,\\ \sum_{j=1}^{n}\lambda_{j}^{t}Z_{ej}^{t}=Z_{eo}^{t},\forall e,t,\\ \begin{array}{c} \sum_{j=1}^{n}\lambda_{j}^{t}Z_{fj}^{t}=Z_{fo}^{t},\forall f,t,\\ \sum_{j=1}^{n}\lambda_{j}^{t}Z_{gj}^{t}=Z_{go}^{t},\forall g,t,\\ \lambda_{j}^{t}\geq 0,\forall j,t,\\ \sum_{j=1}^{n}\lambda_{j}^{t}=1,\forall t; \end{array} \end{array} \end{array}$$ (10)

$$\begin{array}{c} \text{Stage}\;\text{link}\{\begin{array}{c} \sum_{j=1}^{n}\lambda_{j}^{t}Z_{ej}^{t}=\sum_{j=1}^{n}\gamma_{j}^{t}Z_{ej}^{t},\forall e,t,\\ \sum_{j=1}^{n}\lambda_{j}^{t}Z_{fj}^{t}=\sum_{j=1}^{n}\eta_{j}^{t}Z_{fj}^{t},\forall f,t,\\ \sum_{j=1}^{n}\lambda_{j}^{t}Z_{gj}^{t}=\sum_{j=1}^{n}\mu_{j}^{t}Z_{gj}^{t}\forall g,t; \end{array} \end{array}$$ (11)

$$\begin{array}{c} \text{SWT}\;\text{stage}\{\begin{array}{c} \sum_{j=1}^{n}\gamma_{j}^{t}Z_{ej}^{t}=Z_{eo}^{t},\forall e,t,\\ \sum_{j=1}^{n}\gamma_{j}^{t}X_{kj}^{t}=X_{ko}^{t}-s_{k}^{t-},\forall k,t,\\ \sum_{j=1}^{n}\gamma_{j}^{t}Y_{lj}^{t}=Y_{lo}^{t}+s_{l}^{t+},\forall l,t,\\ \begin{array}{c} \gamma_{j}^{t}\geq 0,\forall j,t,\\ \sum_{j=1}^{n}\gamma_{j}^{t}=1,\forall t; \end{array} \end{array} \end{array}$$ (12)

$$\begin{array}{c} \text{WGT}\;\text{stage}\{\begin{array}{c} \sum_{j=1}^{n}\eta_{j}^{t}Z_{fj}^{t}=Z_{fo}^{t},\forall f,t,\\ \sum_{j=1}^{n}\eta_{j}^{t}X_{hj}^{t}=X_{ho}^{t}-s_{h}^{t-},\forall h,t,\\ \sum_{j=1}^{n}\eta_{j}^{t}Y_{pj}^{t}=Y_{po}^{t}-s_{p}^{t-},\forall p,t,\\ \begin{array}{c} \eta_{j}^{t}\geq 0,\forall j,t,\\ \sum_{j=1}^{n}\eta_{j}^{t}=1,\forall t; \end{array} \end{array} \end{array}$$ (13)

$$\begin{array}{c} \text{WWT}\;\text{stage}\{\begin{array}{c} \sum_{j=1}^{n}\mu_{j}^{t}Z_{gj}^{t}=Z_{go}^{t},\forall g,t,\\ \sum_{j=1}^{n}\mu_{j}^{t}C_{mj}^{t-1}=C_{mo}^{t-1},\forall m,t,\\ \sum_{j=1}^{n}\mu_{j}^{t}X_{aj}^{t}=X_{ao}^{t}-s_{a}^{t-},\forall a,t,\\ \begin{array}{c} \sum_{j=1}^{n}\mu_{j}^{t}C_{mj}^{t}=C_{mo}^{t}+s_{m}^{t+},\forall m,t,\\ \sum_{j=1}^{n}\mu_{j}^{t}Y_{qj}^{t}=Y_{qo}^{t}-s_{q}^{t-},\forall q,t\\ \mu_{j}^{t}\geq 0,\forall j,t,\\ \sum_{j=1}^{n}\mu_{j}^{t}=1,\forall t. \end{array} \end{array} \end{array}$$ (14)

$$\begin{array}{c} \text{Period}\;\text{link}\{\sum_{j=1}^{n}\mu_{j}^{t}C_{mj}^{t-1}=\sum_{j=1}^{n}\mu_{j}^{t-1}C_{mj}^{t-1},\forall m,t, \end{array}$$ (15)

$$\begin{array}{c} s_{i}^{t-},s_{r}^{t+},s_{k}^{t-},s_{l}^{t+},s_{h}^{t-},s_{p}^{t-},s_{a}^{t-},s_{m}^{t+},s_{q}^{t-}\geq 0,\forall i,r,k,l,h,p,a,m,q. \end{array}$$ (16)

In the equation above, $s_{i}^{t-},s_{r}^{t+},s_{k}^{t-},s_{l}^{t+},s_{h}^{t-},s_{p}^{t-},s_{a}^{t-},s_{m}^{t+}$ and $s_{q}^{t-}$ represent the slacks value of relevant inputs and outputs, respectively. *β*_1_,*β*_2_,*β*_3_ and *β*_4_ are the weights of P stage, SWT stage, WGT stage and WWT stage, representing the importance in the system. And *α*^*t*^ represents the period weight of period t. Note that $\sum_{d=1}^{4}\beta_{d}=1$, and $\sum_{t=1}^{T}\alpha^{t}=1$, they’re all given exogenously.

Model (9) is a nonlinear programming problem, which can be transformed into a linear programming problem according to [32]’s method. See the Supporting information S1 Equation for the specific linear transformation process.

By solving model (17) in S2 Equation, we can get the optimal solution $\left(\sigma_{j}^{t*},\varsigma_{j}^{t*},\tau_{j}^{t*},\upsilon_{j}^{t*},S_{i}^{t-*},S_{r}^{t+*},S_{k}^{t-*},S_{l}^{t+*},S_{h}^{t-*},S_{p}^{t-*},S_{a}^{t-*},S_{m}^{t+*},S_{q}^{t-*},\phi^{*}\right)$. Then, eco-efficiency($\theta_{o}^{*}$), period efficiency($\theta_{o}^{t*}$), stage efficiency($\theta_{oP}^{*},\theta_{o\text{SWT}}^{*},\theta_{o\text{WGT}}^{*}$, and $\theta_{o\text{WWT}}^{*}$) and period stage efficiency($\theta_{oP}^{t*},\theta_{o\text{SWT}}^{t*},\theta_{o\text{WGT}}^{t*}$ and $\theta_{o\text{WWT}}^{t*}$) are calculated as the Supporting information S2 Equation.

In Eq (26) in S1 Equation, $0<\theta_{o}^{*}\leq 1$. If $\theta_{o}^{*}=1$, the industrial system being evaluated is efficient during this period, otherwise it is inefficient during this period. If $\theta_{o}^{t*}=1$, the industrial system being evaluated is efficient in period t, and if $\theta_{oP}^{*}=1,\theta_{o\text{SWT}}^{*}=1,\theta_{o\text{WGT}}^{*}=1$, and $\theta_{o\text{WWT}}^{*}=1$, the industrial system being evaluated is efficient at the P stage, SWT stage, WGT stage and WWT stage during all the periods. Similarly, if $\theta_{oP}^{t*}=1,\theta_{o\text{SWT}}^{t*}=1,\theta_{o\text{WGT}}^{t*}=1$ and $\theta_{o\text{WWT}}^{t*}=1$, the industrial system at the P stage, SWT stage, WGT stage and WWT stage are efficient during the period t. In terms of model calculation, we mainly use MATLAB R2020a software, supplemented by the results of MaxDEA 7 Ultra software.

## Empirical study

This paper selects 30 provinces in mainland China as the research object. Tibet was excluded for lack of relevant data. The study period spanned from 2011 to 2015. In view of the current situation of environmental pollution in China during the study period and the general trend of planning the development of green economy in the future, the Chinese government issued the Action Plan for air pollution prevention and Control in 2013 and the action Plan for Water pollution Prevention and Control in 2015. However, further investigation is needed to determine whether these policies and plans are having the desired effect. Analysis of the change trend of industrial system efficiency is helpful to find out whether the policy has an impact on the eco-efficiency of industrial system. In this paper, the industrial production system is divided into two stages and four linked subprocesses, which is different from previous studies, such as [20, 33]. It is interesting to find out which sub-processes contributed to the overall system inefficiency during this period.

### Variables

For the P stage (stage 1), this article selects energy, capital, and labor as the input variables. At present, it is almost standard to choose energy, capital, and labor as the input indexes in the P stage, such as [19, 21, 22, 33–35] and so on. Energy is undoubtedly an important element in industrial production. Based on the previous studies, this paper chooses the total industrial energy consumption (TEC) as the energy input indicator. Calculate the total industrial energy consumption according to the provincial energy balance table data, and convert it into standard coal equivalent according to the corresponding standard coefficient [20]. Net fixed assets (NFA) and average annual number of employees (Labor) are proxy variables of capital and labor input in P stage respectively [36]. IAV is the desirable output of stage P. At the same time, stage P also produced three intermediate outputs, namely, WWG, SWG and SDG.

For the stage 2 of the industrial system, it is composed of three divisions, namely SWT stage, WGT stage and WWT stage. These three divisions are parallel structures, together with the P stage, thus forming a hybrid two-stage system. In SWT stage, intermediate product SWG and Solid waste treatment investment (SWTI) are taken as input factors, and the output result is comprehensive utilization of solid waste (desirable output, CUSW). In the WGT stage, input factors include the number of waste gas treatment facilities (WGTF), waste gas treatment investment (WGTI) and intermediate product SDG. The undesirable output is sulfur dioxide emissions (SDE). However, in the WWT stage, the situation is different, mainly due to the presence of carry forward products, treatment capacity of industrial wastewater treatment facilities (WWTFC). The WWTFC generated in the previous year is used as an input to the WWT stage, and WWG, waste water treatment investment (WWTI) and waste water treatment facilities (WWTF) are used as inputs, and wastewater discharge (WWD) is an undesired output. The WWTFC generated in the current year is a desired output. Table 1 lists the input and output variables used in this article.

**Table 1:** Input-output variables.

Stage		Variables	Units
P stage	Inputs	Total energy consumption (TEC)	10-thousand tons of standard coal
		Net fixed assets (NFA)	100-million RMB Yuan
		average annual number of employees (Labor)	10-thousand persons
	Outputs	Industrial added value (IAV)	100-million RMB Yuan
		wastewater generation (WWG)	10-thousand tons
		solid waste generation (SWG)	10-thousand tons
		sulfur dioxide generation (SDG)	10-thousand tons
SWT stage	Inputs	solid waste generation (SWG)	10-thousand tons
		Solid waste treatment investment (SWTI)	10-thousand RMB yuan
	Outputs	comprehensive utilization of solid waste (CUSW)	10-thousand tons
WGT stage	Inputs	sulfur dioxide generation (SDG)	10-thousand tons
		waste gas treatment facilities (WGTF)	sets
		waste gas treatment investment (WGTI)	10-thousand RMB yuan
	Outputs	sulfur dioxide emissions (SDE)	10-thousand tons
WWT stage	Inputs	wastewater generation (WWG)	10-thousand tons
		treatment capacity of industrial wastewater treatment facilities (WWTFC)	10-thousand tons / day
		wastewater treatment investment (WWTI)	10-thousand RMB yuan
		wastewater treatment facilities (WWTF)	sets
	Outputs	treatment capacity of industrial wastewater treatment facilities (WWTFC)	10-thousand tons / day
		wastewater discharge (WWD)	10-thousand tons

### Data

Data sources are mainly from China Industrial Statistical Yearbook (2012–2016), China Energy Statistical Yearbook, China Statistical Yearbook, China Environmental Statistical Yearbook and China Environmental Statistical Annual Report (National Bureau of Statistics of China). Table 2 is the descriptive statistics of the data. According to the descriptive statistical results in Table 2, during the 12th Five Year Plan period, the indicators showing an upward trend in China’s regional industry include TEC, NFA, IAV, CUSW, WGTF and WGTI. Indicators with a certain decline include WWG, SWTI, SDE, WWTFC, WWTI and WWD. The indicators that remain basically stable include SWG, WWTF, SDG and Labor. Therefore, initially, China’s environmental protection policy in the 12th Five Year Plan period has achieved certain results. The average discharge of industrial wastewater is declining, the emission of sulfur dioxide is also showing a downward trend, and the comprehensive utilization of solid waste is also increasing. At the same time, the production of industrial wastewater is declining, while the production of solid waste and sulfur dioxide is also steadily not rising. In short, the data convey a positive message. However, only from the statistical information cannot make a conclusion. We need to further investigate the effectiveness of environmental policy implementation from the perspective of efficiency.

**Table 2:** Descriptive statistics of data.

Variables		2011	2012	2013	2014	2015
TEC	Mean	9519.76	9839.53	10040.77	10034.48	10113.38
	S.D.	6776.94	6911.25	6971.24	6789.89	7021.00
NFA	Mean	7620.88	8544.48	9681.50	10909.13	11512.69
	S.D.	5600.50	6122.66	6850.72	7845.07	8310.72
Labor	Mean	307.99	329.01	326.13	332.30	325.61
	S.D.	324.68	324.33	336.83	340.11	339.35
IAV	Mean	7706.05	7887.71	8394.35	9000.06	8813.73
	S.D.	6788.58	6193.52	6608.49	6784.07	7335.40
WWG	Mean	193500.43	175805.00	164136.33	166590.97	148157.67
	S.D.	186764.96	157573.71	153180.45	161277.87	134160.48
SWG	Mean	10749.07	10955.97	10911.33	10841.23	10889.30
	S.D.	9643.47	9638.67	9295.08	9397.88	9345.74
SDG	Mean	199.49	205.08	210.67	211.03	211.38
	S.D.	130.24	128.10	128.17	139.77	153.89
SWTI	Mean	10454.00	8272.93	4894.20	5020.87	5473.83
	S.D.	14273.25	12737.38	7286.86	7327.01	7772.17
CUSW	Mean	6506.90	6748.50	6863.63	6810.77	6626.57
	S.D.	4891.62	4864.50	4938.01	5093.70	5085.69
WGTF	Mean	7208.00	7522.10	7802.03	8703.13	9686.60
	S.D.	4885.49	5163.34	5359.26	5935.04	6725.53
WGTI	Mean	70541.03	85898.87	213621.40	263082.73	173926.73
	S.D.	58888.34	72150.54	158025.80	256241.65	156311.93
SDE	Mean	67.24	63.72	61.17	58.01	51.89
	S.D.	39.61	37.20	35.71	33.48	29.54
WWTFC	Mean	1046.76	887.13	854.49	843.63	824.03
	S.D.	1274.36	754.02	783.69	755.33	731.48
WWTI	Mean	52560.40	46750.90	41345.77	38158.37	39430.30
	S.D.	55971.97	50618.07	37782.89	37154.28	41037.18
WWTF	Mean	3049.20	2854.77	2675.50	2734.80	2772.83
	S.D.	2501.11	2485.60	2339.81	2323.52	2337.79
WWD	Mean	76946.03	73850.13	69933.30	68433.23	66483.33
	S.D.	63158.96	59455.30	55184.61	52872.77	52147.66

## Results

### Eco and stage efficiency analysis

Before using model (17) in S1 Equation for efficiency analysis, the key step is to determine the period weight and stage weight. For the convenience of analysis, this paper assumes that they are exogenous. According to [8], the period weight increases from front to back, and the period weight of the last period should be the largest, which makes the greatest contribution to the current system. Therefore, the period weights from 2011 to 2015 in this paper are *α*^1^ = 0.1, *α*^2^ = 0.1, *α*^3^ = 0.2, *α*^4^ = 0.3, and *α*^5^ = 0.3, respectively.In terms of stage weight selection, we believe that economy and environment should be equally important. Therefore, *β*_1_ = *β*_2_ = *β*_3_ = *β*_4_ = 0.25. According to the above assumptions, the eco-efficiency of Chinese provincial industrial system can be calculated according to model (17) in S1 Equation.

According to the results in Table 3, we can know that only one region (i.e., Hainan) was evaluated as eco-efficient from 2011 to 2015. The efficiency value of each stage of Hainan industrial system is 1, which further verifies the ecological effectiveness of the industrial system. The ecological environment of Hainan is good. Everyone who has been to Hainan has this feeling. In recent years, Hainan has achieved good ecological benefits under this concept by effectively protecting the ecological environment of Hainan and not seeking economic development at the cost of damaging the ecological environment. Good ecological environment brings high-efficiency tourism economy. During the study period, Hainan accelerated the construction of an international tourism island and implemented the National Strategy of “One Belt, One Road”, actively explored the way forward and took the lead in opening-up its tourism industry. Fujian has the lowest score of eco-efficiency, only 0.8277. There are three efficient stages in Hebei and Shandong, but the overall eco-efficiency is low. During the 12th Five Year Plan period, Hebei’s industrial system is efficient in P stage, SWT stage and WWT stage, but the efficiency of WGT stage is relatively low, only 0.2850, resulting in the eco-efficiency of the whole industrial system of 0.9450. The reason for the low eco-efficiency of Hebei Industrial system comes from the low efficiency of waste gas treatment. Similarly, Shandong operates efficiently in P stage, SWT stage and WGT stage, but the efficiency score of WWT stage is only 0.3511. The reason for the low ecological efficiency of Shandong industrial system comes from the low efficiency of wastewater treatment.

**Table 3:** Chinese regional average efficiency from 2011 to 2015.

Provinces	Eco-efficiency	P efficiency	SWT efficiency	WGT efficiency	WWT efficiency
Beijing	0.9714	1.0000	0.7926	0.7718	0.8814
Tianjin	0.9305	1.0000	1.0000	0.4795	0.4434
Hebei	0.9450	1.0000	1.0000	0.2850	1.0000
Shanxi	0.9591	1.0000	0.7678	0.6761	0.6435
Inner Mongolia	0.9173	1.0000	0.1631	1.0000	0.5116
Liaoning	0.8842	1.0000	0.3693	0.2340	0.8996
Jilin	0.8741	0.9407	0.2259	0.2643	0.7400
Heilongjiang	0.8733	0.9800	0.2847	0.1892	0.8888
Shanghai	0.9057	0.9706	0.8429	0.4048	0.5512
Jiangsu	0.9056	1.0000	0.9985	0.2739	0.3677
Zhejiang	0.8415	1.0000	0.5854	0.1889	0.1178
Anhui	0.9532	0.9105	0.8336	1.0000	0.5485
Fujian	0.8277	1.0000	0.1385	0.1784	0.1708
Jiangxi	0.9073	1.0000	0.2338	0.8738	0.6019
Shandong	0.9541	1.0000	1.0000	1.0000	0.3511
Henan	0.8911	0.9709	0.6368	0.4431	0.4192
Hubei	0.8978	1.0000	0.2511	0.4838	0.6905
Hunan	0.8839	0.9311	0.1250	0.4126	0.8715
Guangdong	0.8619	1.0000	0.0866	0.2308	0.3785
Guangxi	0.8768	1.0000	0.1020	0.5009	0.7574
Hainan	1.0000	1.0000	1.0000	1.0000	1.0000
Chongqing	0.8999	1.0000	0.0919	0.7176	0.5487
Sichuan	0.8475	0.7612	0.3235	0.2985	0.3327
Guizhou	0.9058	1.0000	0.2145	0.6846	0.7749
Yunnan	0.8792	1.0000	0.1405	0.5495	0.5308
Shaanxi	0.8589	0.9639	0.1191	0.3914	0.3892
Gansu	0.9375	1.0000	0.3969	1.0000	0.5850
Qinghai	0.9160	1.0000	0.2047	0.7646	1.0000
Ningxia	0.9216	1.0000	0.1206	0.8913	0.7781
Xinjiang	0.8517	0.8208	0.5392	0.2719	0.2313
Mean	0.9027	0.9750	0.4529	0.5487	0.6002

Through the above analysis, we can draw the following conclusions: first, if and only if P stage, SWT stage, WGT stage and WWT stage operate efficiently, the ecological economy of industrial system is efficient. To make the industrial system have high eco-efficiency, the operation efficiency of four stages must be improved at the same time. second, through our method, we can effectively identify the reasons for the low eco-efficiency of Provincial industrial system.

**Fig 3:**
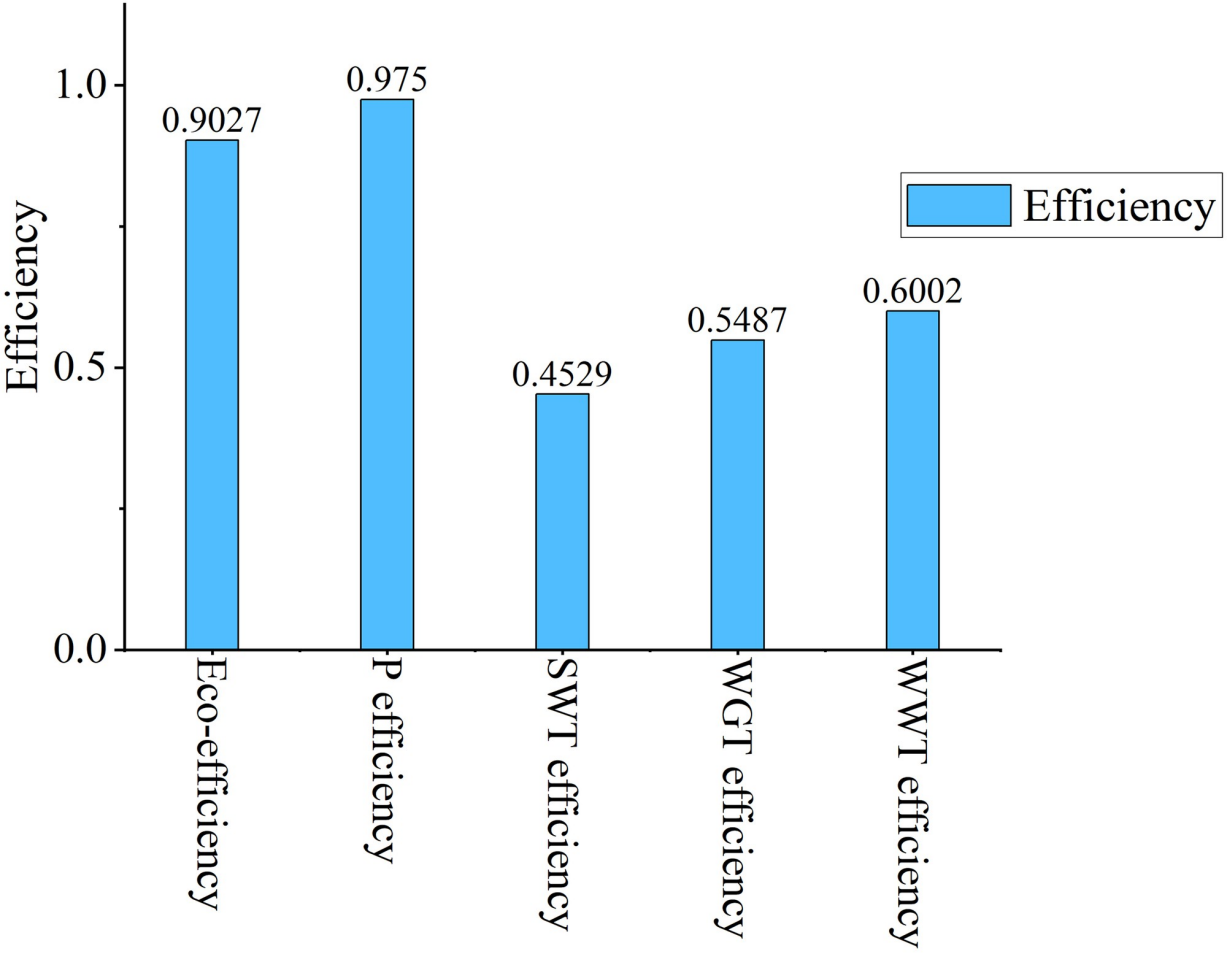
Average efficiency of provincial industrial system.

According to Fig 3, the average efficiency of stage P is the highest during 2011–2015, which is 0.9750. The average efficiency of SWT stage is the lowest, only 0.4529. During the whole survey period, the average eco-efficiency was 0.9027. It can be seen that low average industrial eco-efficiency is mainly caused by the low efficiency of SWT, WGT and WWT stages. Therefore, the solid waste treatment efficiency, waste gas treatment efficiency and wastewater treatment efficiency of China’s Provincial industrial system are still relatively low.

### Period efficiency analysis

According to the model assumptions proposed in the previous section, combined with Eq (27) in S2 Equation, we can calculate the efficiency of the industrial system of each province in each period, and the results are shown in Table 4.

**Table 4:** Regional period efficiency.

Province	2011	2012	2013	2014	2015
Beijing	1.0000	0.6635	0.7919	1.0000	0.8518
Tianjin	0.6057	0.7316	0.8275	0.8654	0.6233
Hebei	0.8605	0.8310	0.8349	0.7964	0.7835
Shanxi	0.6229	0.6951	0.7420	1.0000	0.7992
Inner Mongolia	0.7123	0.6297	0.6159	0.7600	0.6253
Liaoning	0.8220	0.6292	0.4780	0.6520	0.5474
Jilin	0.4469	0.6951	0.5816	0.5404	0.4498
Heilongjiang	0.5844	0.6809	0.5466	0.5595	0.5570
Shanghai	0.7948	0.8125	0.6493	0.6414	0.5639
Jiangsu	0.6402	0.6121	0.6394	0.8167	0.5917
Zhejiang	0.5893	0.5758	0.5135	0.3263	0.3601
Anhui	0.7615	0.8563	1.0000	0.6729	0.8251
Fujian	0.3629	0.4855	0.3469	0.3541	0.3102
Jiangxi	0.6507	0.7216	0.7840	0.7720	0.4584
Shandong	0.7900	0.7948	0.8129	1.0000	0.7911
Henan	0.6938	0.7199	0.4596	0.8304	0.3837
Hubei	0.4819	0.8157	0.6776	0.5735	0.4830
Hunan	0.5836	0.6672	0.6166	0.6261	0.4318
Guangdong	0.4133	0.3524	0.4069	0.3931	0.5542
Guangxi	0.7185	0.6896	0.6486	0.5073	0.3864
Hainan	1.0000	1.0000	1.0000	1.0000	1.0000
Chongqing	0.5534	0.5838	0.6512	0.7532	0.4062
Sichuan	0.3601	0.3606	0.4613	0.3231	0.6397
Guizhou	0.7721	0.6308	0.7178	0.5510	0.6708
Yunnan	0.4713	0.4783	0.6487	0.6926	0.4852
Shaanxi	0.3818	0.3859	0.6005	0.5344	0.4270
Gansu	0.7733	0.5751	0.5980	0.7809	1.0000
Qinghai	0.6822	0.8882	0.7572	0.7656	0.6184
Ningxia	0.6947	0.6563	0.7610	0.7512	0.6241
Xinjiang	0.5194	0.4538	0.3889	0.4922	0.4746
Mean	0.6448	0.6557	0.6519	0.6777	0.5908

**Fig 4:**
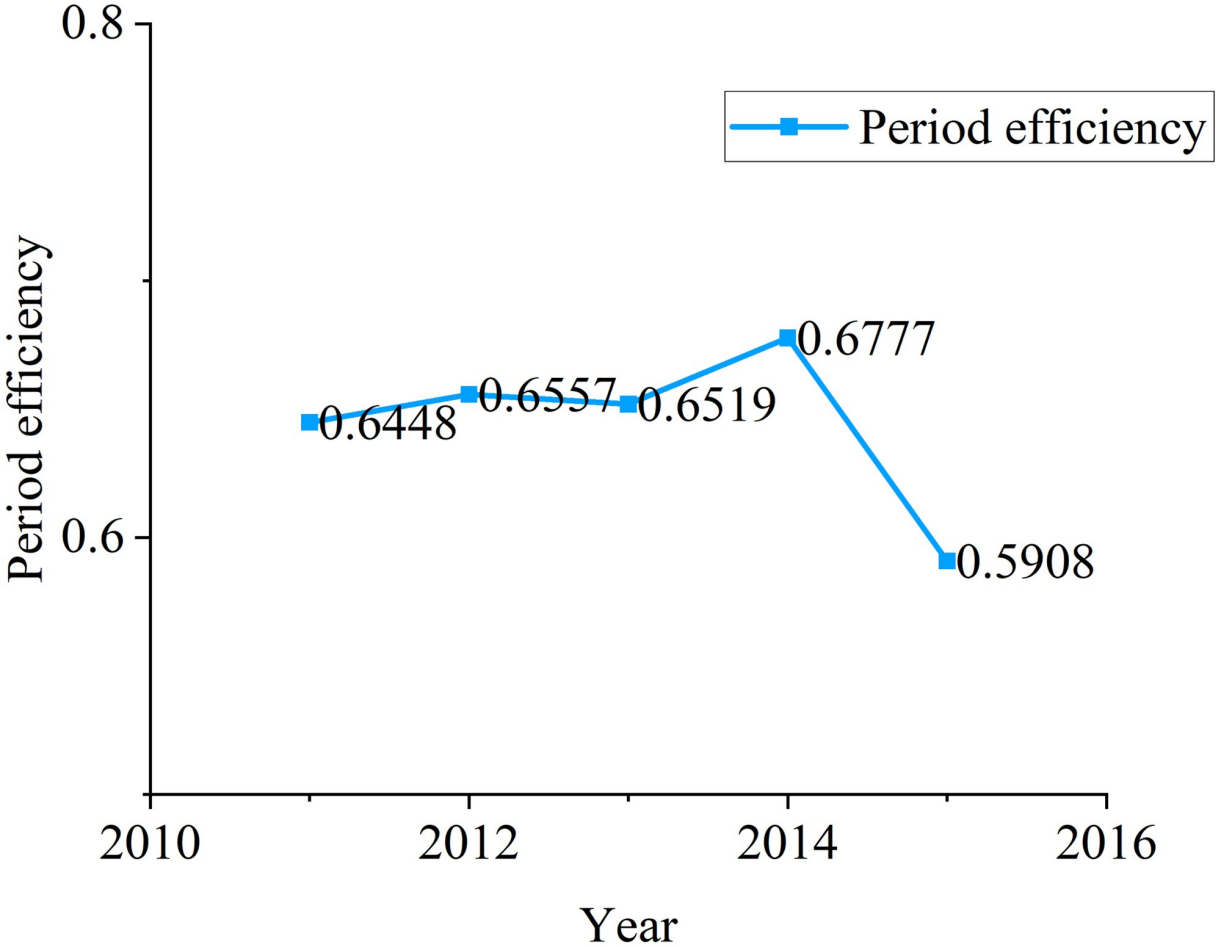
Change trend of period efficiency.

Period efficiency reflects the change trend of eco-efficiency of industrial system with period. According to Fig 4, the eco-efficiency of China’s Provincial industrial system increased and decreased from 2011 to 2015, with a slight decrease, from 0.6448 in 2011 to 0.5908 in 2015. The average period efficiency in 2014 was the highest, 0.6777. Compared with [20]’s research, our method improves the eco-efficiency in each year, which shows that the dynamic research method of hybrid two-stage system can improve the eco-efficiency of Provincial industrial system.

According to Table 4, in 2011, Beijing and Hainan achieved the optimal eco-efficiency, while Sichuan had the lowest eco-efficiency, only 0.3601. Beijing has a good level of economic development and technological innovation, which are conducive to the improvement of industrial eco-efficiency. In 2012, Guangdong had the lowest score of industrial eco-efficiency, which was 0.3524. Hainan is still eco-efficient. From the situation in 2013, Fujian has become the region with the lowest score of eco-efficiency, while Anhui and Hainan have the highest eco-efficiency. In 2014, the eco-efficiency value of Sichuan became the lowest again, 0.3231, and that of Zhejiang was not very good, with a score of 0.3263. In 2015, Fujian and Zhejiang were regions with low eco- efficiency. According to the above analysis, the eco-efficiency of Sichuan and Fujian during the 12th Five Year Plan period is generally not high.

According to the above analysis, during the 12th Five Year Plan period, the industrial eco-efficiency of Sichuan and Fujian is generally low, especially Fujian. The possible causes are as follows: During the 12th Five Year Plan period, Sichuan’s population grew, the process of industrialization, urbanization and agricultural modernization accelerated, the bottleneck constraints on resources and environment further intensified, the contradiction between development and protection became increasingly prominent, and environmental historical problems and new environmental problems were intertwined, which posed a serious threat and impact on the ecological environment, economic and social development of the Three Gorges Reservoir area and even the whole Yangtze River Basin. During the 12th Five Year Plan period, Fujian’s environmental pollution was diversified and complex, showing the characteristics of compound, structural and compressed. The source of pollutants continued to change from industrial and domestic pollution to the coexistence of industry, life and rural areas, and the type of pollutants changed from a single conventional pollutant to a compound of conventional pollutants and new pollutants.

### Period stage efficiency analysis

**Fig 5:**
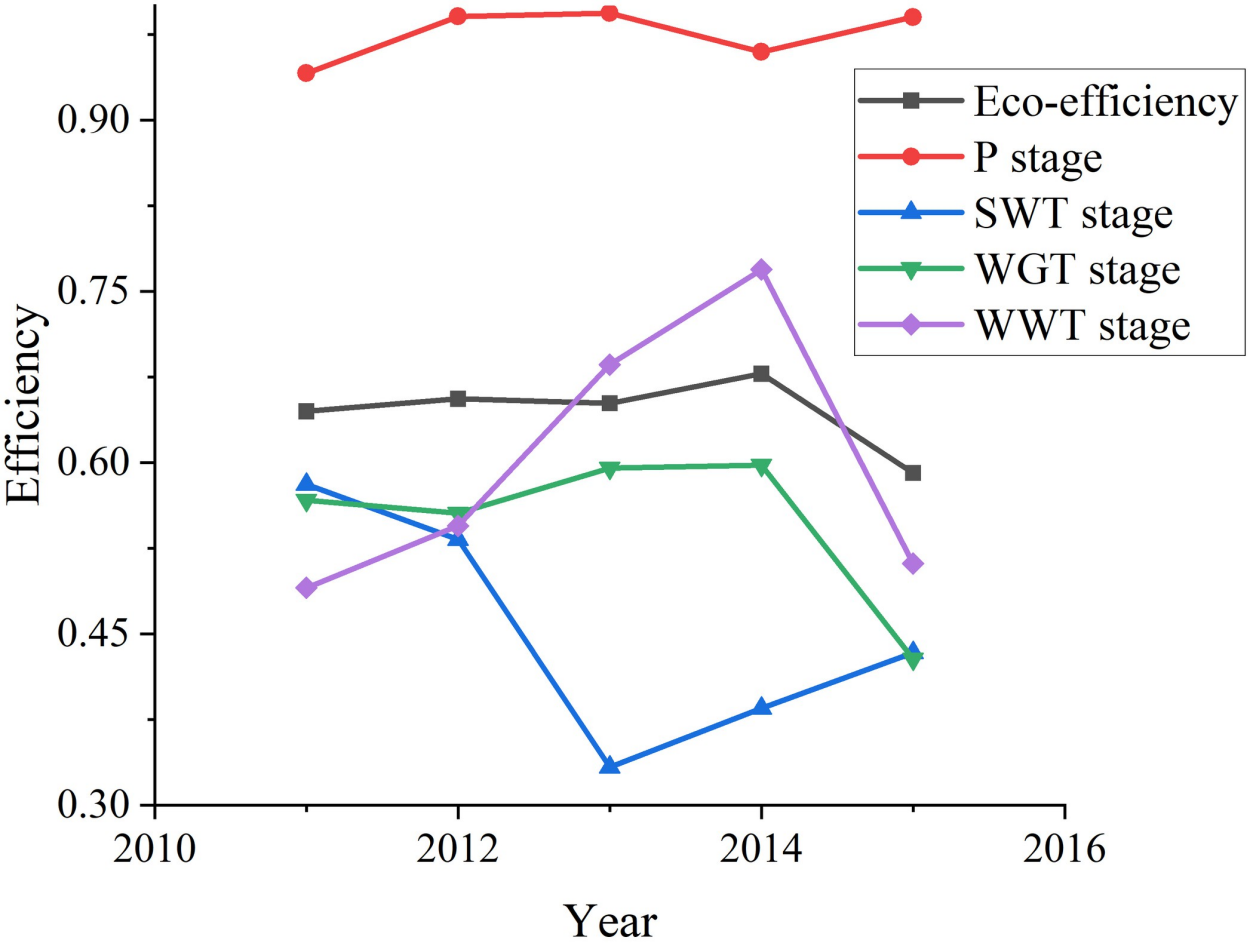
Average period efficiencies during 2011–2015.

The average period stage efficiency is calculated according to the above model assumptions and Eq (32), (33), (34) and (35) in S2 Equation. The results are shown in S1 Table. The trend chart of stage efficiency changing with time as shown in Fig 5 is made.

According to Tables 3 and 4, S1 Table, we know that Hainan industrial system is efficient throughout the study period. During 2011–2015, the efficiency value of each period is 1, and the efficiency of period stage is also 1. This shows that to improve the eco-efficiency of a Provincial industrial system, it is necessary to improve the efficiency of each period and the efficiency value of each period stage at the same time. From 2011 to 2015, the average efficiency of P stage was the highest, reaching 0.9750. The average efficiency of SWT stage is the lowest, 0.4529. This can also be seen in Fig 5.

According to Fig 5, from 2011 to 2015, the efficiency value of P stage showed a steady upward trend, and was generally greater than that of SWT stage, WGT stage and WWT stage. This also reflects the steady improvement of China’s industrial production technology during the 12th Five Year Plan period. Although the efficiency value of WWT stage fluctuates greatly, its efficiency value has not improved much on the overall, from 0.4902 in 2011 to 0.5115 in 2015. The efficiency of SWT stage also fluctuates greatly, but the efficiency has an obvious downward trend, from 0.5809 at the beginning to 0.4335 at the end. The change trend of WGT stage efficiency with period is similar that of system eco-efficiency, which shows that the efficiency of WGT stage has a great impact on the eco-efficiency of industrial system. Moreover, before 2013, except for SWT stage efficiency, system eco-efficiency, P stage efficiency, WWT stage efficiency and WGT stage efficiency kept increasing trend. However, after 2013, the situation was on the contrary. Efficiency began to improve in SWT stage, while the other stages showed a downward trend.

## Discussion

This section focuses on the comparison of provincial industrial system efficiency with and without carry forward products, and the comparative analysis of industrial system efficiency in four major area of China (i.e., eastern area, central area and western area and northeastern area (National Bureau of Statistics of China, 2011).

### Comparison of efficiency with and without carry forward

Without considering the carry forward WWTFC, model (17) in S1 Equation becomes a static model. Table 5 shows the efficiency of the four stages and the eco-efficiency of the system with and without carry forward WWTFC. The average eco-efficiency of the region with carry forward WWTFC (0.9027) was higher than that of the region without WWTFC (0.8821). This suggests that carry forward WWTFC can improve the efficiency of Provincial industrial systems. In addition, this observation was attributed to differences of the WWT efficiency. The average wastewater treatment efficiency with carry forward WWTFC (0.6002) was much higher than that without carry forward WWTFC (0.3691).

**Table 5:** Efficiency results with and without carry forward WWTFC.

Province	With carry forward WWTFC (Ours)					Without carry forward WWTFC				
	Eco	P	SWT	WGT	WWT	Eco	P	SWT	WGT	WWT
Beijing	0.9714	1.0000	0.7926	0.7718	0.8814	0.9504	1.0000	0.7926	0.7718	0.5903
Tianjin	0.9305	1.0000	1.0000	0.4795	0.4434	0.9093	1.0000	1.0000	0.4795	0.2186
Hebei	0.9450	1.0000	1.0000	0.2850	1.0000	0.9450	1.0000	1.0000	0.2850	1.0000
Shanxi	0.9591	1.0000	0.7678	0.6761	0.6435	0.9151	1.0000	0.7678	0.6761	0.2439
Inner Mongolia	0.9173	1.0000	0.1631	1.0000	0.5116	0.8907	1.0000	0.1631	1.0000	0.2750
Liaoning	0.8842	1.0000	0.3693	0.2340	0.8996	0.8674	1.0000	0.3693	0.2340	0.5716
Jilin	0.8741	0.9407	0.2259	0.2643	0.7400	0.8541	0.9407	0.2259	0.2643	0.4360
Heilongjiang	0.8733	0.9800	0.2847	0.1892	0.8888	0.8492	0.9800	0.2847	0.1892	0.5462
Shanghai	0.9057	0.9706	0.8429	0.4048	0.5512	0.8983	0.9706	0.8429	0.4048	0.5157
Jiangsu	0.9056	1.0000	0.9985	0.2739	0.3677	0.8837	1.0000	0.9985	0.2739	0.1818
Zhejiang	0.8415	1.0000	0.5854	0.1889	0.1178	0.8381	1.0000	0.5854	0.1889	0.1025
Anhui	0.9532	0.9105	0.8336	1.0000	0.5485	0.9364	0.9105	0.8336	1.0000	0.3829
Fujian	0.8277	1.0000	0.1385	0.1784	0.1708	0.8221	1.0000	0.1385	0.1784	0.1455
Jiangxi	0.9073	1.0000	0.2338	0.8738	0.6019	0.8792	1.0000	0.2338	0.8738	0.2933
Shandong	0.9541	1.0000	1.0000	1.0000	0.3511	0.9336	1.0000	1.0000	1.0000	0.1816
Henan	0.8911	0.9709	0.6368	0.4431	0.4192	0.8631	0.9709	0.6368	0.4431	0.1830
Hubei	0.8978	1.0000	0.2511	0.4838	0.6905	0.8787	1.0000	0.2511	0.4719	0.4219
Hunan	0.8839	0.9311	0.1250	0.4126	0.8715	0.8508	0.9311	0.1250	0.4126	0.3199
Guangdong	0.8619	1.0000	0.0866	0.2308	0.3785	0.8327	1.0000	0.0866	0.2308	0.1501
Guangxi	0.8768	1.0000	0.1020	0.5009	0.7574	0.8583	1.0000	0.1020	0.5009	0.4000
Hainan	1.0000	1.0000	1.0000	1.0000	1.0000	1.0000	1.0000	1.0000	1.0000	1.0000
Chongqing	0.8999	1.0000	0.0919	0.7176	0.5487	0.8619	1.0000	0.0919	0.7176	0.1760
Sichuan	0.8475	0.7612	0.3235	0.2985	0.3327	0.8315	0.7612	0.3235	0.2985	0.2013
Guizhou	0.9058	1.0000	0.2145	0.6846	0.7749	0.8813	1.0000	0.2145	0.6846	0.4751
Yunnan	0.8792	1.0000	0.1405	0.5495	0.5308	0.8544	1.0000	0.1405	0.5495	0.2168
Shaanxi	0.8589	0.9639	0.1191	0.3914	0.3892	0.8335	0.9639	0.1191	0.3914	0.1090
Gansu	0.9375	1.0000	0.3969	1.0000	0.5850	0.9002	1.0000	0.3969	1.0000	0.2218
Qinghai	0.9160	1.0000	0.2047	0.7646	1.0000	0.9160	1.0000	0.2047	0.7646	1.0000
Ningxia	0.9216	1.0000	0.1206	0.8913	0.7781	0.8827	1.0000	0.1128	0.8913	0.3195
Xinjiang	0.8517	0.8208	0.5392	0.2719	0.2313	0.8462	0.8208	0.5392	0.2719	0.1935
Mean	0.9027	0.9750	0.4529	0.5487	0.6002	0.8821	0.9750	0.4527	0.5483	0.3691

**Fig 6:**
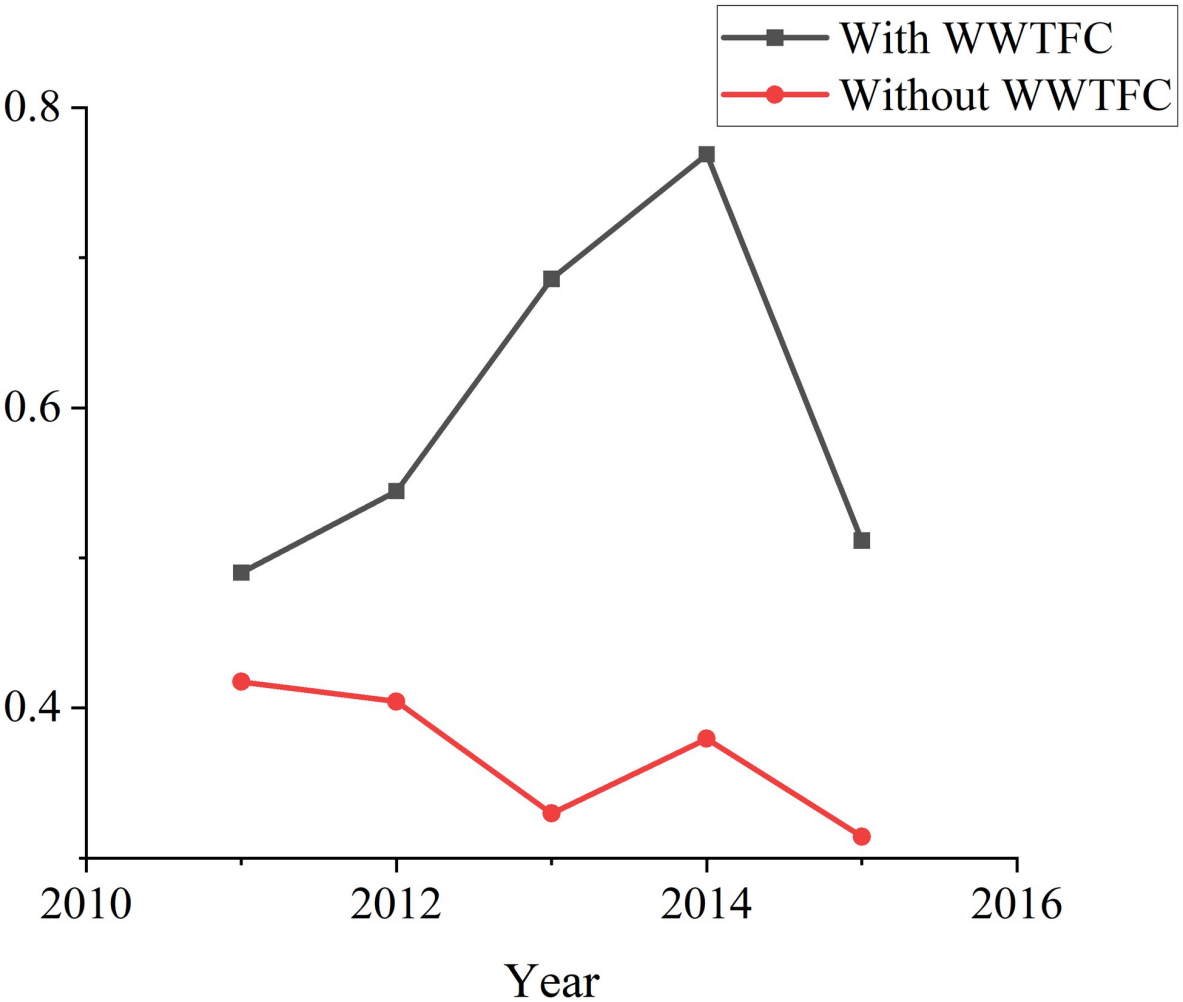
Average WWT efficiency during 2011–2015.

Further comparison of carry forward WWTFC is shown in Fig 6. From 2011 to 2015, the efficiency of wastewater treatment with carry forward WWTFC increased, while that without WWTFC decreased. For each year, the efficiency of wastewater treatment using WWTFC is greater than that without WWTFC. This shows that the efficiency of wastewater treatment in Provincial industrial systems can be measured more accurately considering the dynamics.

The results showed that: (1) WWTFC from 2011 to 2015 had a significant impact on the improvement of eco-efficiency of Provincial industrial systems in China; (2) Provincial industrial systems with high wastewater treatment efficiency are more likely to have high eco-efficiency. When measuring the eco-efficiency of Provincial industrial system, the dynamic hybrid two-stage DEA method is adopted to model WWTFC as carry forward variable, which improves the discrimination ability of system eco-efficiency, especially the efficiency of wastewater treatment. This is similar to [37], who believed that the inclusion of carry forward variables in the two-stage DDEA model would increase the discrimination ability of overall performance. Therefore, in order to improve the eco-efficiency of provincial industrial system, local governments can consider establishing incentive mechanism to mobilize the enthusiasm of industrial enterprises and improve the efficiency of WWTFC and wastewater treatment stage. For example, implement environmental protection investment and financing policies and preferential tax policies to encourage the research and development of cleaner production technologies.

### Efficiency comparisons of four areas

The study takes 31 regions in mainland China as the research object, and divides them into four areas: Eastern area, Central area, Western area, and Northeastern areas (National Bureau of Statistics of China, 2011). Table 6 lists details for the 31 regions. The study shows that there are obvious geographical characteristics of Provincial industrial system efficiency in China.

**Table 6:** Areas in China.

Areas	Regions
Eastern area	Beijing, Tianjin, Hebei, Shanghai, Jiangsu, Zhejiang, Fujian, Shandong, Guangdong, and Hainan
Central area	Shanxi, Anhui, Jiangxi, Henan, Hubei, and Hunan
Western area	Inner Mongolia, Guangxi, Chongqing, Sichuan, Guizhou, Yunnan, Tibet, Shaanxi, Gansu, Qinghai, Ningxia, and Xinjiang
Northeastern area	Liaoning, Jilin, and Heilongjiang

**Fig 7:**
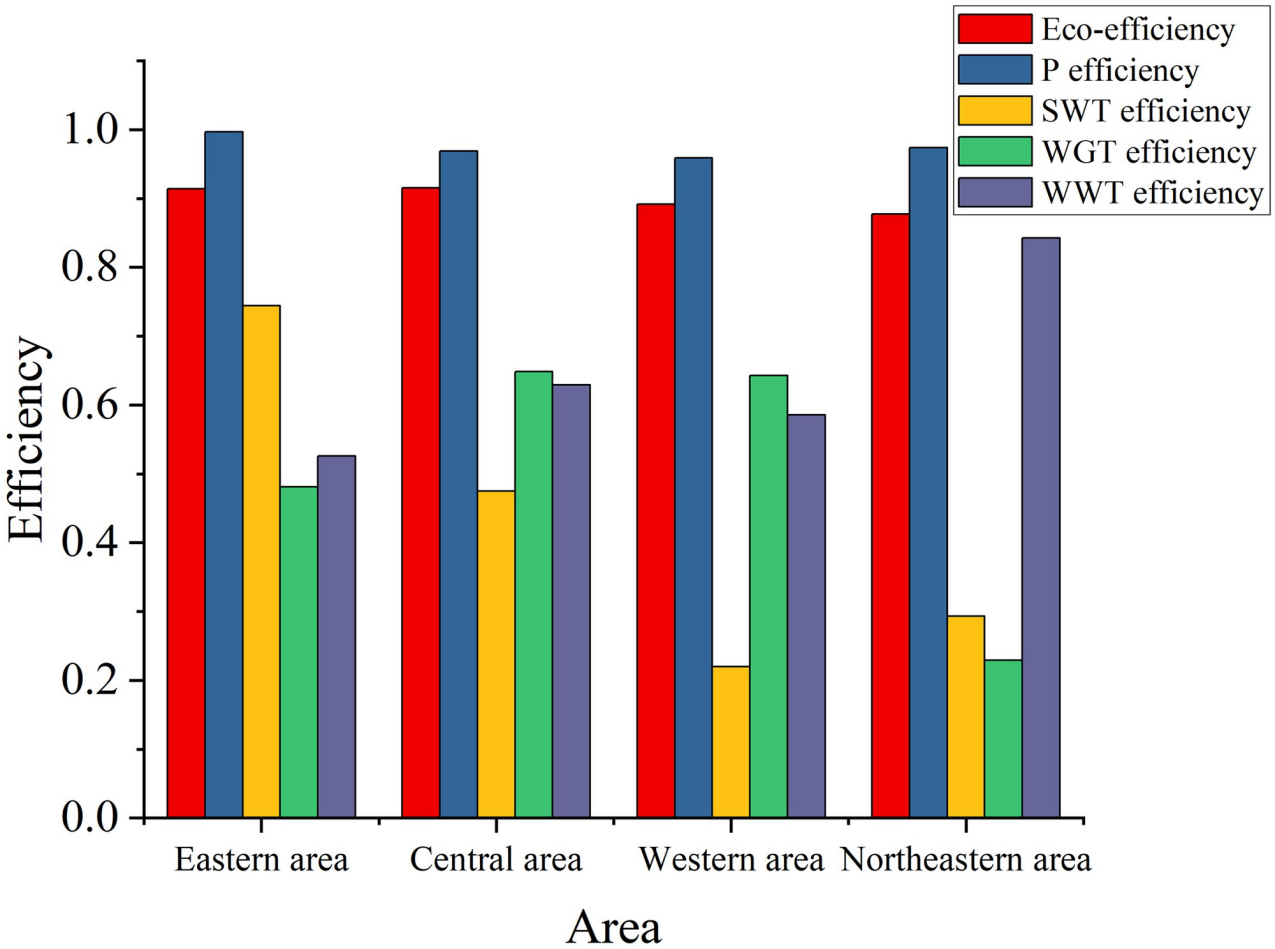
Average eco-efficiency and stage efficiencies.

The average eco-efficiency and stage efficiencies are depicted in Fig 7. As can be seen from Fig 7, the average eco-efficiency of Eastern and Central areas is higher (0.9143, 0.9154), while the average eco-efficiency of Northeastern area is the lowest (0.8772). This observation shows that the industrial system in the Eastern and Central areas performs better on average than the rest of China. In terms of stage efficiency, the Eastern area has the highest production stage and solid waste treatment stage efficiency (0.9971, 0.7444). The Central area has the highest waste gas treatment efficiency (0.6482). The average efficiency of production stage and solid waste treatment stage was the lowest in Western China (0.9587, 0.2196). The waste gas treatment efficiency in Northeast China was the lowest (0.2292), and the waste water treatment efficiency was the highest (0.8428). These results show that Eastern and Central regions performed well in all four stages. The Eastern and Central areas of China are relatively developed regions. Complete infrastructure, abundant capital, abundant human resources and strong government support.

The eco-efficiency of the industrial system in Western area ranked third (0.8920), the waste gas treatment stage efficiency ranked second (0.6428) and waste water treatment stage efficiency ranked third (0.5854). This shows that under the guidance of the national Western development strategy, the industrial system in the Western area has achieved certain achievements, remarkable achievements in social and economic development, rapid economic growth and sound industrial system. Therefore, in order to improve the operational efficiency of the industrial system in western area, the Chinese government should continue to make efforts, continue to implement the Western development strategy, increase investment in R&D, and improve the production efficiency and comprehensive utilization rate of solid waste through technological upgrading.

The study also found that the wastewater treatment stage efficiency in Northeast area was the highest at 0.8428, but the eco-efficiency of the industrial system and the waste gas treatment stage efficiency were the lowest at 0.8772 and 0.2292. This finding suggests that the Northeast is doing better than other regions at the wastewater treatment stage. However, it performs worse than other regions in the overall ecology and waste gas treatment stage of the industrial system. This suggests that the inefficiencies in the Northeast area are mainly due to the waste gas treatment stage. This is in line with the actual situation in Northeast area of China. The industrialization foundation of Northeast is relatively old, and emission reduction measures are relatively backward. This leads to a relatively low eco-efficiency in the area. That means making industrial emissions more efficient and increasing investment in advanced emissions-reduction technologies.

According to the above results of efficiency analysis in different areas, the following policy implications can be drawn: firstly, Compared with the Eastern, Central and Northeastern areas, the average efficiency of industrial production and solid waste treatment in the Western area was the lowest during the 12th Five-Year Plan period. Therefore, the industrial sectors in the Western area should continue to improve the industrial infrastructure, expand the channels for investment and formulate more competitive talent introduction policies. We will actively strengthen communication and contact with the local government, strive for further support from the government, continuously increase r&d input in industrial production and industrial waste treatment technology (especially solid waste treatment), and actively explore a new mode of “industry-university-research” cooperation. Secondly, the overall level of industrial eco-efficiency in Western China is relatively high, which indicates that China’s Western development strategy has achieved certain results. To continue to improve the operational efficiency of the industrial system in the Western region, the Chinese government should continue its efforts, continue to implement the strategy of developing the western region, increase investment in R & D, and improve the efficiency of industrial production and the comprehensive utilization rate of solid waste through technological upgrading. Thirdly, Northeast China is China’s old industrial base, but its industrialization base is still old and industrial pollution reduction technology is relatively backward, resulting in the average ecological efficiency of the region is relatively low. Therefore, in order to improve the level of industrial ecological efficiency in northeast China and revitalize the old industrial base in an all-round way, it is necessary to speed up the transformation of industrial economic development mode, actively eliminate backward production capacity and speed up the transformation of old and new driving forces of industrial development under the guidance of policies. The green and sustainable industrial development should be emphasized, new technologies should lead new development, and trans-regional cooperation should be actively sought to jointly cope with the difficulties of industrial economic development, so as to realize the sustainable and healthy development of industrial economy.

## Conclusion

According to SBM method, this paper proposes a dynamic hybrid two-stage model for eco-efficiency evaluation of Chinese provincial industrial system from 2011 to 2015. Chinese provincial industrial system can be regarded as a dynamic two-stage structure composed of production stage and emission reduction stage. The emission reduction stage also includes three parallel sub-stages, namely solid waste treatment stage, waste gas treatment stage and waste water treatment stage. The WWTFC of the wastewater treatment process in the provincial industrial system is used as a carry forward variable to connect the two consecutive periods of the emission reduction stage. This method not only considers the internal structure of the provincial industrial system, but also considers the dynamic effect of carry forward variables, which provides useful information for the reasons for the low efficiency of the provincial industrial system. This paper investigates 30 provincial industrial systems in China. According to the research results, we can draw conclusions as follows.

Firstly, the provincial industrial system in China from 2011 to 2015 is generally eco-inefficient, which mainly comes from the SWT and WGT stages. Secondly, from 2011 to 2014, China’s regional average eco-efficiency showed a trend of steady improvement, which also shows that the Chinese government has achieved certain results in implementing environmental and economic policies. It declined slightly in 2015, which may be caused by the fact that WWTFC carry forward from the previous period was not carry forward to the current period or the delayed effect of environmental and economic policies. Thirdly, carry forward WWTFC in Provincial industrial systems can improve the efficiency of industrial systems, especially the efficiency of WWT stage. In the WWT stage, provincial industrial systems with high efficiency are more likely to perform well in ecology. Fourth, the reasons for the inefficiency of industrial systems in different geographical areas are different. The inefficiency of the eastern area is mainly caused by the WGT and WWT stages. In contrast, inefficiencies in the central and western areas are mainly due to the SWT stage. The low efficiency in northeast area is mainly due to the low efficiency in WGT and SWT stages.

In this study, WWTFC generated in the current period will be used as the technical basis for industrial wastewater treatment in the next stage. However, technology transfer in the treatment of industrial waste gas and solid waste is not considered. In addition, there is no research on the interaction between stages within the industrial system, which is a focus that may need to be studied.

## Supporting information

S1 Appendix Relevant source data. In this paper, the use of relevant source data are available from the National Bureau of Statistics official website http://www.stats.gov.cn/english/ to download.
(XLSX)

S1 Table Period stage efficiency. The average period stage efficiency is calculated according to the above model assumptions and Eq (32), (33), (34) and (35) in S2 Equation.
(XLSX)

S1 Equation Linear transformation. (PDF)

S2 Equation (PDF)
