# Supplementary material for: Eco-efficiency evaluation of Chinese provincial industrial system: A dynamic hybrid two-stage DEA approach
Source: PLoS One. 2022 Aug 5;17(8):e0272633. doi: 10.1371/journal.pone.0272633 (PMC9355237; doi:10.1371/journal.pone.0272633)
Supplement: S1 Equation — (PDF) [file pone.0272633.s003.pdf]

S1 Equation. Linear transformation.

Setting $\varphi = \frac{1}{\sum_{t=1}^{T} \alpha^t \left[\beta_1 \left(1+\frac{1}{R}\sum_{r=1}^{R}\frac{s_r^{t+}}{Y_{ro}^t}\right)+\beta_2\left(1+\frac{1}{L}\sum_{l=1}^{L}\frac{s_l^{t+}}{Y_{lo}^t}\right)+\beta_4\left(1+\frac{1}{M}\sum_{m=1}^{M}\frac{s_m^{t+}}{C_{mo}^t}\right)\right]}$, $\sigma_j^t = \varphi\lambda_j^t$, $\varsigma_j^t = \varphi\gamma_j^t$, $\tau_j^t = \varphi\eta_j^t$, $\upsilon_j^t = \varphi\mu_j^t$, $S_i^{t-} = \varphi s_i^{t-}$, $S_r^{t+} = \varphi s_r^{t+}$, $S_k^{t-} = \varphi s_k^{t-}$, $S_l^{t+} = \varphi s_l^{t+}$, $S_h^{t-} = \varphi s_h^{t-}$, $S_p^{t-} = \varphi s_p^{t-}$, $S_a^{t-} = \varphi s_a^{t-}$, $S_a^{t-} = \varphi s_a^{t-}$, $S_m^{t+} = \varphi s_m^{t+}$, $S_m^{t+} = \varphi s_m^{t+}$, $S_q^{t-} = \varphi s_q^{t-}$, $\forall i, r, k, l, h, p, a, m, q$.. Therefore, model (9) will become

$$\theta_o = \min \sum_{t=1}^{T} \alpha^t \left[\begin{array}{l} \beta_1\left(\varphi - \frac{1}{I}\sum_{i=1}^{I}\frac{S_i^{t-}}{X_{io}^t}\right) + \beta_2\left(\varphi - \frac{1}{K}\sum_{k=1}^{K}\frac{S_k^{t-}}{X_{ko}^t}\right) \\ +\beta_3\left(\varphi - \frac{1}{H+P}\left(\sum_{h=1}^{H}\frac{S_h^{t-}}{X_{ho}^t} + \sum_{p=1}^{P}\frac{S_p^{t-}}{Y_{po}^t}\right)\right) \\ +\beta_4\left(\varphi - \frac{1}{A+Q}\left(\sum_{a=1}^{A}\frac{S_a^{t-}}{X_{ao}^t} + \sum_{q=1}^{Q}\frac{S_q^{t-}}{Y_{qo}^t}\right)\right) \end{array} \right] \tag{17}$$

$$\text{P stage}\begin{cases} \sum_{j=1}^{n}\sigma_j^t X_{ij}^t = \varphi X_{io}^t - S_i^{t-}, \forall i, t, \\ \sum_{j=1}^{n}\sigma_j^t Y_{rj}^t = \varphi Y_{ro}^t + S_r^{t+}, \forall r, t, \\ \sum_{j=1}^{n}\sigma_j^t Z_{ej}^t = \varphi Z_{eo}^t, \forall e, t, \\ \sum_{j=1}^{n}\sigma_j^t Z_{fj}^t = \varphi Z_{fo}^t, \forall f, t, \\ \sum_{j=1}^{n}\sigma_j^t Z_{gj}^t = \varphi Z_{go}^t, \forall g, t, \\ \sigma_j^t \geq 0, \forall j, t, \\ \sum_{j=1}^{n}\sigma_j^t = \varphi, \forall t; \end{cases} \tag{18}$$

$$\text{Stage link}\begin{cases} \sum_{j=1}^{n}\sigma_j^t Z_{ej}^t = \sum_{j=1}^{n}\varsigma_j^t Z_{ej}^t, \forall e, t, \\ \sum_{j=1}^{n}\sigma_j^t Z_{fj}^t = \sum_{j=1}^{n}\tau_j^t Z_{fj}^t, \forall f, t, \\ \sum_{j=1}^{n}\sigma_j^t Z_{gj}^t = \sum_{j=1}^{n}\upsilon_j^t Z_{gj}^t \forall g, t; \end{cases} \tag{19}$$

$$\text{SWT stage}\begin{cases} \sum_{j=1}^{n}\varsigma_j^t Z_{ej}^t = \varsigma Z_{eo}^t, \forall e, t, \\ \sum_{j=1}^{n}\varsigma_j^t X_{kj}^t = \varphi X_{ko}^t - S_k^{t-}, \forall k, t, \\ \sum_{j=1}^{n}\varsigma_j^t Y_{lj}^t = \varphi Y_{lo}^t + S_l^{t+}, \forall l, t, \\ \varsigma_j^t \geq 0, \forall j, t, \\ \sum_{j=1}^{n}\varsigma_j^t = \varphi, \forall t; \end{cases} \tag{20}$$

$$\text{WGT stage}\begin{cases} \sum_{j=1}^{n}\tau_j^t Z_{fj}^t = \varphi Z_{fo}^t, \forall f, t, \\ \sum_{j=1}^{n}\tau_j^t X_{hj}^t = \varphi X_{ho}^t - S_h^{t-}, \forall h, t, \\ \sum_{j=1}^{n}\tau_j^t Y_{pj}^t = \varphi Y_{po}^t - S_p^{t-}, \forall p, t, \\ \tau_j^t \geq 0, \forall j, t, \\ \sum_{j=1}^{n}\tau_j^t = \varphi, \forall t; \end{cases} \tag{21}$$

$$
\text{WWT stage}\begin{cases}
\sum_{j=1}^{n} \upsilon_j^t Z_{gj}^t = \varphi Z_{go}^t, \forall g, t,\\
\sum_{j=1}^{n} \upsilon_j^t C_{mj}^{t-1} = \varphi C_{mo}^{t-1}, \forall m, t,\\
\sum_{j=1}^{n} \upsilon_j^t X_{aj}^t = \varphi X_{ao}^t - S_a^{t-}, \forall a, t,\\
\sum_{j=1}^{n} \upsilon_j^t C_{mj}^t = \varphi C_{mo}^t + S_m^{t+}, \forall m, t,\\
\sum_{j=1}^{n} \upsilon_j^t Y_{qj}^t = \varphi Y_{qo}^t - S_q^{t-}, \forall q, t\\
\upsilon_j^t \geq 0, \forall j, t,\\
\sum_{j=1}^{n} \upsilon_j^t = \varphi, \forall t.
\end{cases} \tag{22}
$$

$$
\text{Period link}\begin{cases}\sum_{j=1}^{n} \upsilon_j^t C_{mj}^{t-1} = \sum_{j=1}^{n} \upsilon_j^{t-1} C_{mj}^{t-1}, \forall m, t,\end{cases} \tag{23}
$$

$$
\sum_{t=1}^{T} \alpha^t \left[\beta_1 \left(\varphi + \frac{1}{R}\sum_{r=1}^{R} \frac{S_r^{t+}}{Y_{ro}^t}\right) + \beta_2 \left(\varphi + \frac{1}{L}\sum_{l=1}^{L} \frac{S_l^{t+}}{Y_{lo}^t}\right) + \beta_4 \left(\varphi + \frac{1}{M}\sum_{m=1}^{M} \frac{S_m^{t+}}{C_{mo}^t}\right)\right] = 1 \tag{24}
$$

$$
S_i^{t-}, S_r^{t+}, S_k^{t-}, S_l^{t+}, S_h^{t-}, S_p^{t-}, S_a^{t-}, S_m^{t+}, S_q^{t-} \geq 0, \forall i, r, k, l, h, p, a, m, q. \tag{25}
$$
