# Supplementary material for: Eco-efficiency evaluation of Chinese provincial industrial system: A dynamic hybrid two-stage DEA approach
Source: PLoS One. 2022 Aug 5;17(8):e0272633. doi: 10.1371/journal.pone.0272633 (PMC9355237; doi:10.1371/journal.pone.0272633)
Supplement: S2 Equation — (PDF) [file pone.0272633.s004.pdf]

S2 Equation.

$$\theta_o^* = \min \sum_{t=1}^{T} \alpha^t \left[\begin{array}{l} \beta_1 \left(\varphi^* - \frac{1}{I} \sum\limits_{i=1}^{I} \frac{S_i^{t-*}}{X_{io}^t} \right) + \beta_2 \left(\varphi^* - \frac{1}{K} \sum\limits_{k=1}^{K} \frac{S_k^{t-*}}{X_{ko}^t} \right) \\ + \beta_3 \left(\varphi^* - \frac{1}{H+P} \left(\sum\limits_{h=1}^{H} \frac{S_h^{t-*}}{X_{ho}^t} + \sum\limits_{p=1}^{P} \frac{S_p^{t-*}}{Y_{po}^t} \right) \right) \\ + \beta_4 \left(\varphi^* - \frac{1}{A+Q} \left(\sum\limits_{a=1}^{A} \frac{S_a^{t-*}}{X_{ao}^t} + \sum\limits_{q=1}^{Q} \frac{S_q^{t-*}}{Y_{qo}^t} \right) \right) \end{array} \right] \tag{26}$$

$$\theta_o^{t*} = \frac{\begin{array}{c} \beta_1 \left(\varphi^* - \frac{1}{I} \sum\limits_{i=1}^{I} \frac{S_i^{t-*}}{X_{io}^t} \right) + \beta_2 \left(\varphi^* - \frac{1}{K} \sum\limits_{k=1}^{K} \frac{S_k^{t-*}}{X_{ko}^t} \right) \\ + \beta_3 \left(\varphi^* - \frac{1}{H+P} \left(\sum\limits_{h=1}^{H} \frac{S_h^{t-*}}{X_{ho}^t} + \sum\limits_{p=1}^{P} \frac{S_p^{t-*}}{Y_{po}^t} \right) \right) \\ + \beta_4 \left(\varphi^* - \frac{1}{A+Q} \left(\sum\limits_{a=1}^{A} \frac{S_a^{t-*}}{X_{ao}^t} + \sum\limits_{q=1}^{Q} \frac{S_q^{t-*}}{Y_{qo}^t} \right) \right) \end{array}}{\beta_1 \left(\varphi^* + \frac{1}{R} \sum\limits_{r=1}^{R} \frac{S_r^{t+*}}{Y_{ro}^t} \right) + \beta_2 \left(\varphi^* + \frac{1}{L} \sum\limits_{l=1}^{L} \frac{S_l^{t+*}}{Y_{lo}^t} \right) + \beta_4 \left(\varphi^* + \frac{1}{M} \sum\limits_{m=1}^{M} \frac{S_m^{t+*}}{C_{mo}^t} \right)} \tag{27}$$

$$\theta_{o\mathrm{P}}^* = \frac{\sum\limits_{t=1}^{T} \alpha^t \left(\varphi^* - \frac{1}{I} \sum\limits_{i=1}^{I} \frac{S_i^{t-*}}{X_{io}^t} \right)}{\sum\limits_{t=1}^{T} \alpha^t \left(\varphi^* + \frac{1}{R} \sum\limits_{r=1}^{R} \frac{S_r^{t+*}}{Y_{ro}^t} \right)} \tag{28}$$

$$\theta^*_{o\mathrm{SWT}} = \frac{\sum\limits_{t=1}^{T} \alpha^t \left(\varphi^* - \frac{1}{K} \sum\limits_{k=1}^{K} \frac{S_k^{t-*}}{X_{ko}^t} \right)}{\sum\limits_{t=1}^{T} \alpha^t \left(\varphi^* + \frac{1}{L} \sum\limits_{l=1}^{L} \frac{S_l^{t+*}}{Y_{lo}^t} \right)} \tag{29}$$

$$\theta^*_{o\mathrm{WGT}} = \sum_{t=1}^{T} \alpha^t \left(\varphi^* - \frac{1}{H+P} \left(\sum_{h=1}^{H} \frac{S_h^{t-*}}{X_{ho}^t} + \sum_{p=1}^{P} \frac{S_p^{t-*}}{Y_{po}^t} \right) \right) \tag{30}$$

$$\theta^*_{o\mathrm{WWT}} = \frac{\sum\limits_{t=1}^{T} \alpha^t \left(\varphi^* - \frac{1}{A+Q} \left(\sum\limits_{a=1}^{A} \frac{S_a^{t-*}}{X_{ao}^t} + \sum\limits_{q=1}^{Q} \frac{S_q^{t-*}}{Y_{qo}^t} \right) \right)}{\sum\limits_{t=1}^{T} \alpha^t \left(\varphi^* + \frac{1}{M} \sum\limits_{m=1}^{M} \frac{S_m^{t+*}}{C_{mo}^t} \right)} \tag{31}$$

$$\theta^{t*}_{o\mathrm{P}} = \frac{\varphi^* - \frac{1}{I} \sum\limits_{i=1}^{I} \frac{S_i^{t-*}}{X_{io}^t}}{\varphi^* + \frac{1}{R} \sum\limits_{r=1}^{R} \frac{S_r^{t+*}}{Y_{ro}^t}} \tag{32}$$

$$\theta^{t*}_{o\mathrm{SWT}} = \frac{\varphi^* - \frac{1}{K} \sum\limits_{k=1}^{K} \frac{S_k^{t-*}}{X_{ko}^t}}{\varphi^* + \frac{1}{L} \sum\limits_{l=1}^{L} \frac{S_l^{t+*}}{Y_{lo}^t}} \tag{33}$$

$$\theta^{t*}_{o\mathrm{WGT}} = \varphi^* - \frac{1}{H+P} \left(\sum_{h=1}^{H} \frac{S_h^{t-*}}{X_{ho}^t} + \sum_{p=1}^{P} \frac{S_p^{t-*}}{Y_{po}^t} \right) \tag{34}$$

$$\theta^{t*}_{o\mathrm{WWT}} = \frac{\varphi^* - \frac{1}{A+Q} \left(\sum\limits_{a=1}^{A} \frac{S_a^{t-*}}{X_{ao}^t} + \sum\limits_{q=1}^{Q} \frac{S_q^{t-*}}{Y_{qo}^t} \right)}{\varphi^* + \frac{1}{M} \sum\limits_{m=1}^{M} \frac{S_m^{t+*}}{C_{mo}^t}} \tag{35}$$
